# Myocardial Hemorrhage After Acute Reperfused ST-Segment–Elevation Myocardial Infarction: Relation to Microvascular Obstruction and Prognostic Significance

**DOI:** 10.1161/CIRCIMAGING.115.004148

**Published:** 2016-01-19

**Authors:** David Carrick, Caroline Haig, Nadeem Ahmed, Margaret McEntegart, Mark C. Petrie, Hany Eteiba, Stuart Hood, Stuart Watkins, M. Mitchell Lindsay, Andrew Davie, Ahmed Mahrous, Ify Mordi, Samuli Rauhalammi, Naveed Sattar, Paul Welsh, Aleksandra Radjenovic, Ian Ford, Keith G. Oldroyd, Colin Berry

**Affiliations:** From the BHF Glasgow Cardiovascular Research Centre, Institute of Cardiovascular and Medical Sciences (D.C., N.A., I.M., S.R., N.S., P.W., A.R., K.G.O., C.B.) and Robertson Centre for Biostatistics (C.H., I.F.), University of Glasgow, Glasgow, United Kingdom; Golden Jubilee National Hospital, Clydebank, United Kingdom (D.C., M.M., M.C.P., H.E., S.H., S.W., M.M.L., A.D., A.M., C.B.).

**Keywords:** hemorrhage, magnetic resonance imaging, myocardial infarction, myocardial reperfusion, prognosis

## Abstract

**Background—:**

The success of coronary reperfusion therapy in ST-segment–elevation myocardial infarction (MI) is commonly limited by failure to restore microvascular perfusion.

**Methods and Results—:**

We performed a prospective cohort study in patients with reperfused ST-segment–elevation MI who underwent cardiac magnetic resonance 2 days (n=286) and 6 months (n=228) post MI. A serial imaging time-course study was also performed (n=30 participants; 4 cardiac magnetic resonance scans): 4 to 12 hours, 2 days, 10 days, and 7 months post reperfusion. Myocardial hemorrhage was taken to represent a hypointense infarct core with a T2* value of <20 ms. Microvascular obstruction was assessed with late gadolinium enhancement. Adverse remodeling was defined as an increase in left ventricular end-diastolic volume ≥20% at 6 months. Cardiovascular death or heart failure events post discharge were assessed during follow-up. Two hundred forty-five patients had evaluable T2* data (mean±age, 58 [11] years; 76% men). Myocardial hemorrhage 2 days post MI was associated with clinical characteristics indicative of MI severity and inflammation. Myocardial hemorrhage was a multivariable associate of adverse remodeling (odds ratio [95% confidence interval]: 2.64 [1.07–6.49]; *P*=0.035). Ten (4%) patients had a cardiovascular cause of death or experienced a heart failure event post discharge, and myocardial hemorrhage, but not microvascular obstruction, was associated with this composite adverse outcome (hazard ratio, 5.89; 95% confidence interval, 1.25–27.74; *P*=0.025), including after adjustment for baseline left ventricular end-diastolic volume. In the serial imaging time-course study, myocardial hemorrhage occurred in 7 (23%), 13 (43%), 11 (33%), and 4 (13%) patients 4 to 12 hours, 2 days, 10 days, and 7 months post reperfusion. The amount of hemorrhage (median [interquartile range], 7.0 [4.9–7.5]; % left ventricular mass) peaked on day 2 (*P*<0.001), whereas microvascular obstruction decreased with time post reperfusion.

**Conclusions—:**

Myocardial hemorrhage and microvascular obstruction follow distinct time courses post ST-segment–elevation MI. Myocardial hemorrhage was more closely associated with adverse outcomes than microvascular obstruction.

**Clinical Trial Registration—:**

URL: http://www.clinicaltrials.gov. Unique identifier: NCT02072850.

## Abstract 2

Supplemental Digital Content is available in the text.

The success of emergency coronary reperfusion therapy in ST-segment–elevation myocardial infarction (STEMI) is commonly limited by failed tissue perfusion.^1^ This disconnect is mainly because of 2 pathologies: microvascular obstruction^2^ and intramyocardial hemorrhage.^3^ On the basis of morphological^2^ and functional studies,^4^ microvascular obstruction may have structural and functional components,^5^ reflecting irreversible (ie, endothelial disruption) and reversible (eg, microvascular spasm and extrinsic edema) components. Myocardial hemorrhage reflects the aggregation and extravasation of erythrocytes^3,6,7^ and is a manifestation of severe microvascular injury. Knowledge of the temporal evolution of these pathologies post STEMI would be relevant to inform the use of novel therapies intended to limit the progression of reperfusion injury and also to inform the timing of cardiac magnetic resonance (CMR) for surrogate end point assessment in clinical trials.

**See Editorial by Arheden**

**See Clinical Perspective**

T2*-weighted CMR is the reference diagnostic method for myocardial hemorrhage in vivo^8,9^; however, technical issues have constrained T2* imaging in clinical practice. To date, the larger cohort studies of myocardial hemorrhage in patients with STEMI have not used T2* imaging,^7,10–13^ implying the lack of specificity. T2*-mapping has been used in smaller STEMI cohorts.^14–16^ These limitations are clinically relevant because of the uncertainties around the detection and clinical significance of myocardial hemorrhage and its relationships with microvascular obstruction. Myocardial hemorrhage is associated with adverse remodeling,^10,12–14,16^ persistent left ventricular (LV) systolic dysfunction,^13,17^ late arrhythmic risk,^15^ and adverse clinical outcome^18^; however, myocardial hemorrhage may^13^ or may not^10–12^ have prognostic significance beyond microvascular obstruction.

In this study, we aimed to (1) specifically detect myocardial hemorrhage using T2* mapping in a large relatively unselected STEMI population and re-evaluate its pathophysiology and prognostic significance, (2) study the temporal evolution of myocardial hemorrhage with serial CMR early after reperfusion, and (3) assess the temporal relationships between myocardial hemorrhage versus microvascular obstruction.

We used quantitative T2* mapping that potentially offers increased accuracy for the detection of myocardial hemorrhage than T2-weighted methods^8^ because T2*-relaxation times are measured directly^19–21^ and is less sensitive to the effects of edema,^22^ which may mask hemorrhage.

## Methods

### Study Population and STEMI Management

We performed a prospective CMR cohort study of myocardial hemorrhage in acute reperfused STEMI survivors treated by emergency percutaneous coronary intervention (PCI), in a single-regional cardiac center between July 14, 2011, and November 22, 2012. Three hundred twenty-four patients with STEMI provided written informed consent. This study was approved by the National Research Ethics Service (reference, 10-S0703-28).

Acute STEMI management followed contemporary guidelines^23^ (Methods section in the Data Supplement). The study was publically registered (ClinicalTrials.gov identifier: NCT02072850).

### CMR Acquisition

CMR was performed on a Siemens MAGNETOM Avanto (Erlangen, Germany) 1.5-Tesla scanner with a 12-element phased array cardiac surface coil 2 days and 6 months post MI.^24^ CMR was planned for 2 days and 6 months in all participants.

The imaging protocol^25,26^ (Methods section in the Data Supplement) included cine CMR with steady-state free precession, T2* mapping, T2 mapping,^19^ and delayed-enhancement phase-sensitive inversion-recovery pulse sequences.^27^ To respect the word count, the CMR methods are provided in the Data Supplement.

### Serial Imaging Substudy

Thirty patients with STEMI underwent serial CMR to characterize the evolution of myocardial hemorrhage by T2 and T2* quantification and evaluate the temporal relationship with microvascular obstruction. Each patient was imaged at 4 time points, with the identical imaging protocol as above: 4 to 12 hours, 2 days, 10 days, and 6 to 7 months post reperfusion.

### Healthy Volunteers

CMR was also performed in 50 healthy volunteers of similar age and sex to obtain local reference values for myocardial T2 and T2* (Data Supplement). Patients and healthy volunteers underwent the same imaging protocol except that healthy volunteers aged <45 years did not receive gadolinium. The coefficients of variation for native T2 and T2* were also measured (Results section in the Data Supplement).

### CMR Analyses

The CMR analyses are described in the Methods section in the Data Supplement.

#### T2 and T2*—Standardized Measurements in Myocardial Regions of Interest

LV contours were delineated with computer-assisted planimetry on the raw T2* image and the last corresponding T2 raw image, with echo time of 55 ms^28^ (Methods section in the Data Supplement).

#### Myocardial Hemorrhage

On the T2* maps, a region of reduced signal intensity within the infarcted area, with a T2* value of <20 ms^14,16,20^ was considered to confirm the presence of myocardial hemorrhage.

#### Infarct Definition and Size

The myocardial mass of late gadolinium (g) was quantified using computer-assisted planimetry^24^ (Methods section in the Data Supplement).

#### Area At Risk

Area at risk was defined as LV myocardium with pixel values (T2) >2 SDs from remote myocardium.^29–32^

#### Myocardial Salvage

Myocardial salvage was calculated by subtraction of percent infarct size from percent area at risk.^29–32^ The myocardial salvage index was calculated by dividing the myocardial salvage area by the initial area at risk.

#### Adverse Remodeling

Adverse remodeling was defined as an increase in LV end-diastolic volume ≥20% at 6 months from baseline.^33^

### Electrocardiogram

A 12-lead ECG was obtained before coronary reperfusion and 60 minutes post procedure (Methods section in the Data Supplement). ECG evidence of reperfusion injury was taken as persistence of ST-segment–elevation resolution post procedure.

### Prespecified Health Outcomes

We prespecified adverse health outcomes that are pathophysiologically linked with STEMI. The primary composite outcome was cardiovascular death or first heart failure hospitalization post discharge. Events that occurred during the index hospitalization were not counted. The serious adverse events were independently assessed by an accredited cardiologist who was not a member of the research team. The serious adverse events were defined according to standard criteria^25,26^ and categorized as having occurred either during the index admission or post discharge. All study participants were followed up by patient contacts through telephone calls, clinic visits, and review of the electronic medical records for a minimum of 18 months after discharge.

### Statistical Analyses

The sample size calculation and statistical methods are described in the Methods section in the Data Supplement. Categorical variables are expressed as number and percentage of patients. Most continuous variables followed a normal distribution and are therefore presented as means together with SD. Those variables that did not follow a normal distribution are presented as medians with interquartile range. Differences between groups were assessed using 1-way ANOVA, Kruskal–Wallis test, or Fisher exact test where appropriate. Agreement was assessed with Bland–Altman plots. For repeated measures, linear mixed effects modeling, with subject ID as a random factor, was used. Binary logistic regression models were used to identify predictors of adverse remodeling at 6-month follow-up. Linear regressions were used to assess relationships in models with continuous responses. Where backward stepwise variable selection was used for either logistic or linear models, the Akaike information criterion was used a measure of the relative quality of the models, and the model with the minimum Akaike information criterion value was reported.

Cox proportional hazards methods were used to identify potential clinical predictors of cardiovascular death/heart failure events, including patient characteristics, CMR findings, and myocardial hemorrhage. The assumption of proportional hazards was verified by fitting an interaction between time and predictor. All statistical analyses were carried out using R version 2.15.1 or SAS version 9.3, or later versions of these programs. A *P* value of >0.05 indicates the absence of evidence for a statistically significant association.

## Results

**Figure 1.**
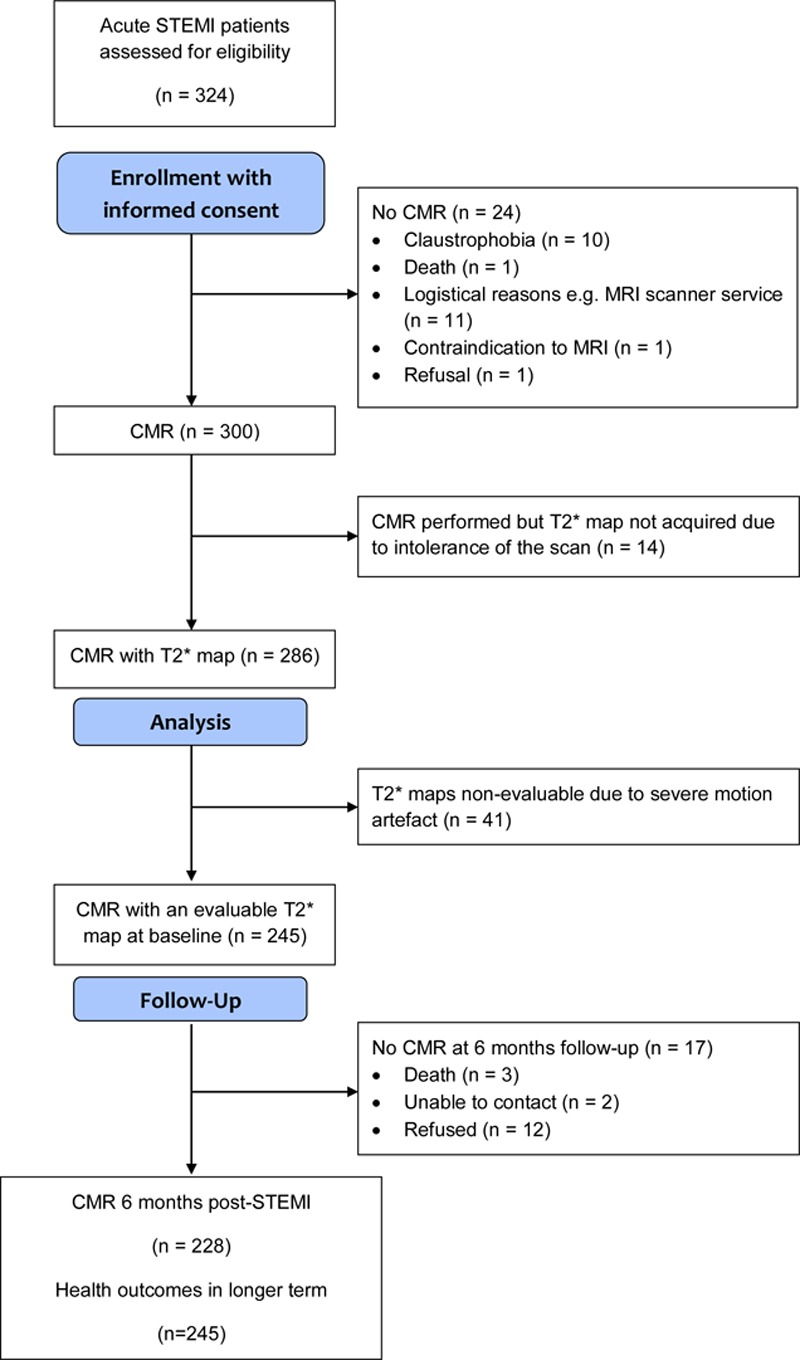
Study flow diagram. CMR indicates cardiac magnetic resonance; MRI, magnetic resonance imaging, and STEMI, ST-segment–elevation myocardial infarction.

Of 324 patients with STEMI referred for emergency PCI, 300 underwent serial CMR 2.1±1.8 days and 6 months after hospital admission (Figure 1). Two hundred eighty-six patients with STEMI had T2* maps acquired. Two hundred forty-five (86%) patients had evaluable myocardial T2* data (Figure 1), and all of these patients had evaluable T2 maps. CMR follow-up at 6 months was achieved in 228 (93%) of the patients with T2* mapping performed, and all (n=245) patients had health outcomes assessed at minimum of 18 months after enrollment.

### Patient Characteristics

The clinical characteristics of the patients (n=245), categorized according to the presence or the absence of myocardial hemorrhage and microvascular obstruction 2 days post reperfusion, are shown in Table 1. One hundred one (41%) patients had myocardial hemorrhage as specifically revealed by T2* maps. Male sex, anterior infarction, thrombolysis in MI flow ≤1 before PCI, Killip heart failure class >2 at presentation, and inflammation were more common in patients with myocardial hemorrhage. Patients with myocardial hemorrhage had less resolution of ST-segment–elevation post PCI. At discharge, angiotensin-converting enzyme inhibitor and β-blocker therapies were prescribed in 237 (97%) and 233 (95%) of patients, respectively.

**Table 1.**
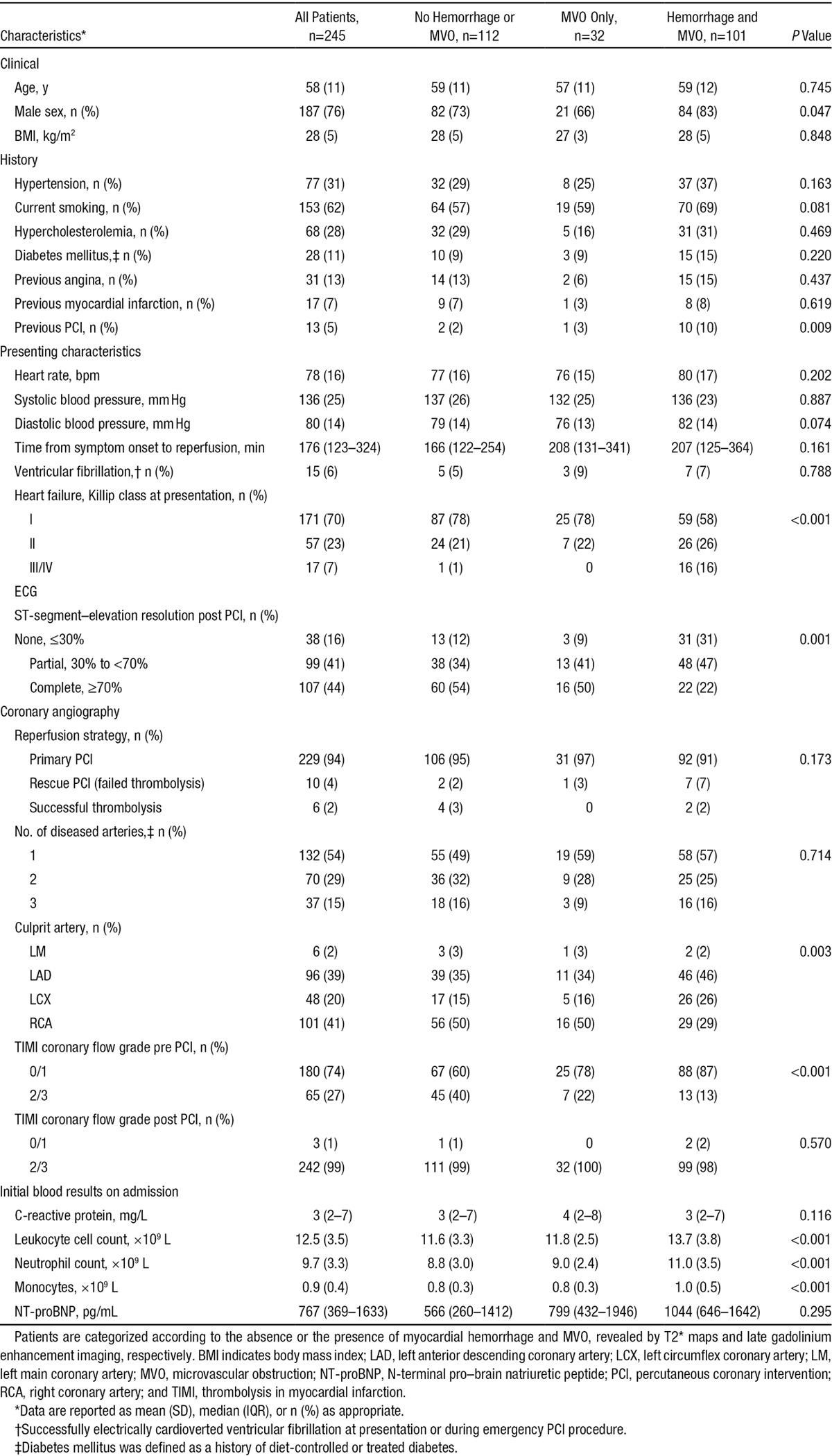
Clinical and Angiographic Characteristics of 245 Patients With Acute ST-Segment–Elevation Myocardial Infarction Who Had Cardiac Magnetic Resonance at Baseline, Including Evaluable T2* Maps

### Myocardial Hemorrhage and Associations With Clinical Characteristics and Inflammation

In stepwise logistic regression using Akaike information criterion, myocardial hemorrhage was associated with sex, smoking, history of previous PCI, thrombolysis in MI coronary flow grade at initial angiography, ECG evidence of reperfusion injury, Killip class, and markers of inflammation, including peak monocyte count and peak neutrophil count (all *P*<0.03; Table I in the Data Supplement).

### Myocardial Hemorrhage Is Associated With Myocardial Infarct Characteristics

**Figure 2.**
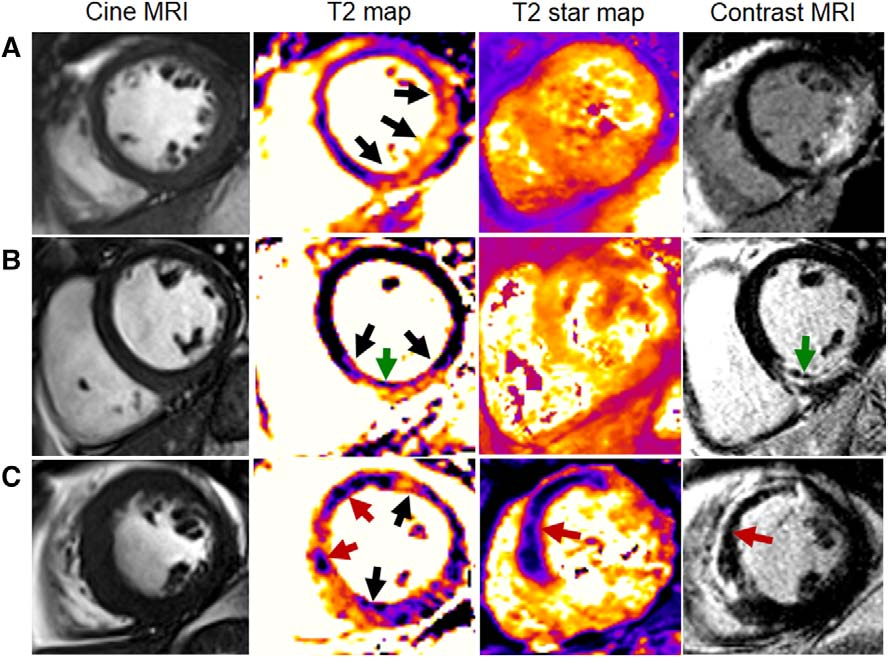
Three patients with acute ST-segment–elevation myocardial infarction treated by primary percutaneous coronary intervention (PCI) using the same antithrombotic strategies. Each patient had normal thrombolysis in myocardial infarction grade 3 flow at the end of PCI. Cardiac magnetic resonance imaging was performed 2 days post reperfusion. **A**, Patient with no evidence of myocardial hemorrhage or microvascular obstruction. **B**, Patient with T2-hypointense core and microvascular obstruction, in the absence of hemorrhage. **C**, Patient with myocardial hemorrhage (Results section in the Data Supplement).

The CMR findings at 2 days post MI and 6 months later are shown in Table 2. Clinical cases are shown in Figure 2. Compared with patients without myocardial hemorrhage, patients with myocardial hemorrhage had a larger LV mass, larger LV volumes, and lower LV ejection fractions early post MI and at 6 months. The initial area at risk, infarct size, and microvascular obstruction were also larger, and there was less myocardial salvage in patients with myocardial hemorrhage, (*P*<0.001; Table 2).

**Table 2.**
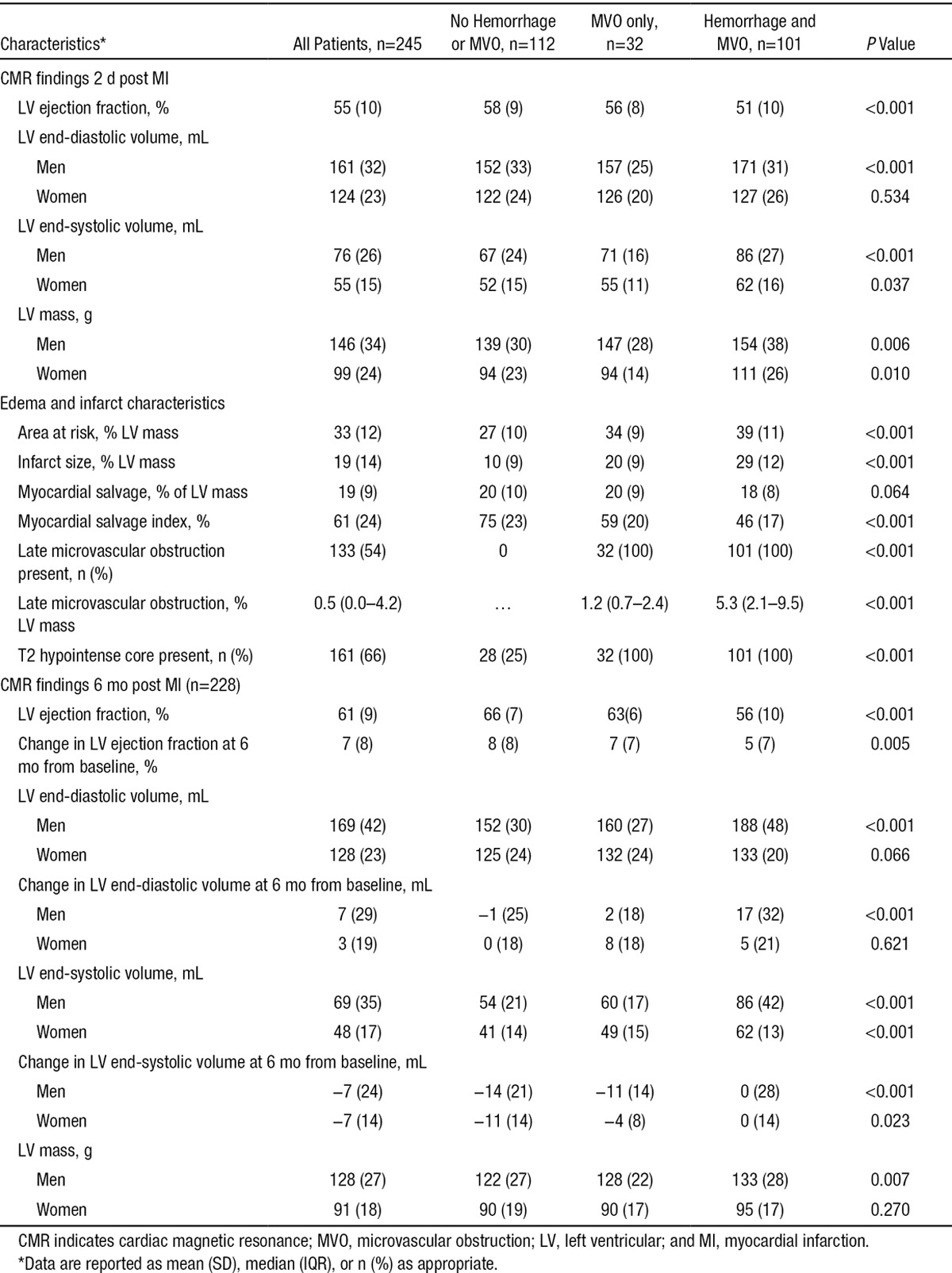
Baseline and 6-Month CMR Findings in 245 Patients With ST-Segment–Elevation Myocardial Infarction According to the Presence or the Absence of Myocardial Hemorrhage and Microvascular Obstruction

#### Comparison of Myocardial Hemorrhage (T2* Core), T2 Hypointense Core, and Microvascular Obstruction

A hypointense infarct core was detected with T2 mapping in 161 (66%) patients with STEMI. Microvascular obstruction with early and late gadolinium enhancement CMR was revealed in 151 (62%) and 133 (51%) patients, respectively. All patients with myocardial hemorrhage, as defined by T2* imaging, had late microvascular obstruction and a hypointense core on T2 imaging. By contrast, 32 (13%) patients had late microvascular obstruction in the absence of myocardial hemorrhage, and all of these patients had a hypointense core on T2 imaging. Twenty-eight (11%) patients had a T2 hypointense core without evidence of late microvascular obstruction or myocardial hemorrhage.

The results of intraobserver and interobserver agreement of T2 and T2* core measurements are shown in Results section in the Data Supplement.

### Temporal Evolution of Myocardial Hemorrhage and Microvascular Obstruction From Acute Reperfusion Through to 6 Months

Thirty patients with STEMI underwent serial imaging on 4 successive occasions, and their clinical characteristics (Table II in the Data Supplement) and CMR findings (Tables III and IV in the Data Supplement) were similar to those of the whole cohort. The imaging time points post reperfusion were (mean±SD) 8.6±3.1 hours, 2.9±1.5 days, 9.6±2.3 days, and 213±27 days (100% compliance).

**Figure 3.**
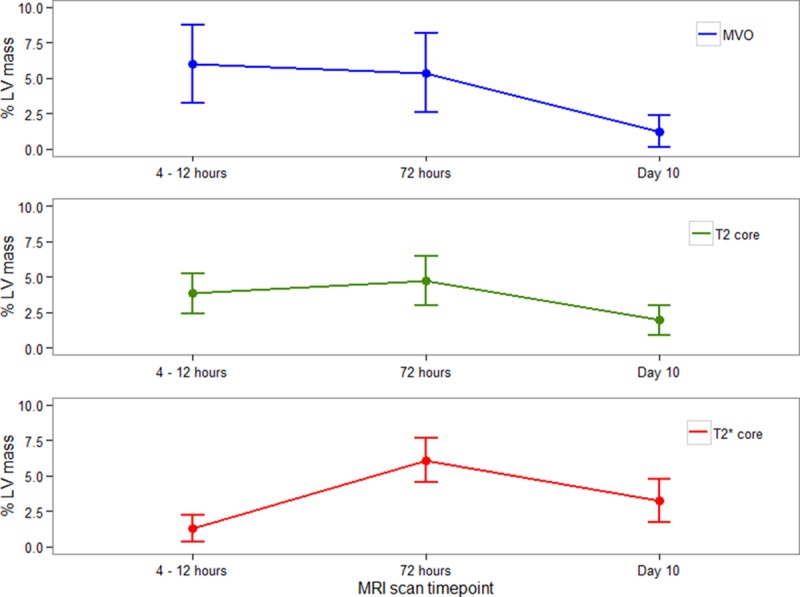
Temporal evolution of the extent of microvascular obstruction (MVO; **top**, blue line), the hypointense core revealed by T2 mapping (**middle**, green line), and myocardial hemorrhage (**bottom**, red line) revealed by T2* mapping according to time from coronary reperfusion in 28 ST-segment–elevation myocardial infarction survivors. Data are presented as median (interquartile range) percentage of left ventricular (LV) mass. The amount of MVO and T2-hypointense core were calculated using full LV coverage, whereas the amount of myocardial hemorrhage was derived from 3 scans from the basal, mid, and apical slice acquisitions. *P* values were obtained from linear mixed effects model with subject as a random factor. MRI indicates magnetic resonance imaging.

Intramyocardial hemorrhage occurred in 7 (23%), 13 (43%), 11 (33%), and 4 (13%) patients versus microvascular obstruction in 18 (60%), 17 (57%), 10 (33%), and 0 patients at 4 to 12 hours, 2 days, 10 days, and 7 months, respectively. The amount of microvascular obstruction (% LV mass) in patients with hemorrhagic infarction was at its greatest at 4 to 12 hours post reperfusion and remained similar at day 2 CMR and then reduced by day 10 (*P*<0.001; Figure 3; Table V in the Data Supplement). By contrast, the amount of myocardial hemorrhage progressively increased from 4 to 12 hours with a peak at day 2 and decreased by day 10 (*P*<0.001; Figure 3). The amount of T2-hypointense core (% LV mass) followed a similar time course to hemorrhage (*P*<0.001; Figure 3). At 7 months, 4 (13%) patients had evidence of persisting hemorrhage, but none of the patients had microvascular obstruction.

#### Persistence of Microvascular Obstruction in Relation to the Presence of Myocardial Hemorrhage

Microvascular obstruction revealed by late gadolinium enhancement resolved by day 10 in 8 (44%) patients; 2 (25%) of whom had evidence of myocardial hemorrhage. Microvascular obstruction persisted at day 10 in 10 (56%) patients, and all (100%) of these patients had evidence of myocardial hemorrhage.

### Myocardial Hemorrhage and Adverse Remodeling at 6 Months

At 6 months, LV end-diastolic volume increased on average (SD) by 6 (27) mL in 224 patients with evaluable data. The average increase in LV end-diastolic volume at 6 months was greater in patients with myocardial hemorrhage compared with those without (15 [30] versus 1 [22]; *P*<0.001). Adverse remodeling, defined as an increase in LV end-diastolic volume by ≥20%, occurred in 28 (13%) patients. The presence of myocardial hemorrhage (binary) was a multivariable associate of adverse remodeling, independent of baseline LV end-diastolic volume (odds ratio [95% confidence interval], 2.64 [1.07–6.49]; *P*=0.035; Table VI in the Data Supplement). In multivariable regression, T2* core (continuous, ms) was not significantly associated with adverse remodeling.

#### Myocardial Hemorrhage, Microvascular Obstruction, T2-Hypointense Core, and LV Outcomes at 6 Months

The relationships for the presence of myocardial hemorrhage, T2 map core, and microvascular obstruction for LV outcomes, including LV end-diastolic volumes and LV ejection fraction, are shown in Table 3. Myocardial hemorrhage is consistently associated with worse LV outcomes 6 months post MI.

**Table 3.**
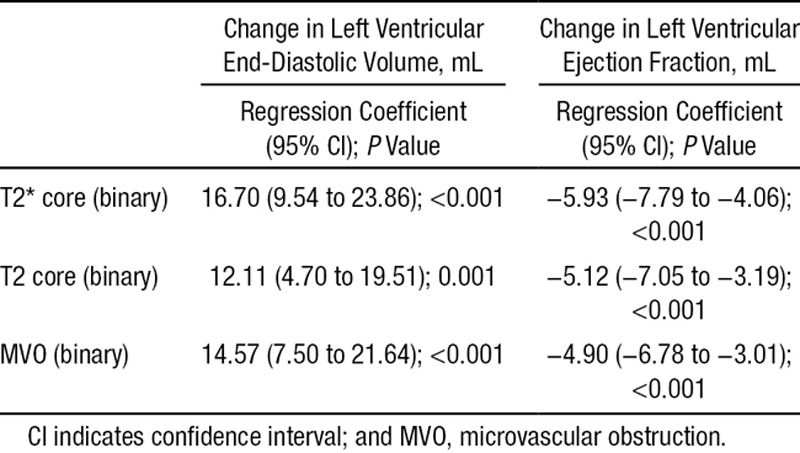
Coefficients, 95% CIs, and P Values From Linear Regression for the Associations of the Presence of Myocardial Hemorrhage (T2* Core) and T2-Map Infarct Core and MVO, With Changes in Left Ventricular Volume and Ejection Fraction at 7 Months, Adjusted for Baseline

#### Myocardial Hemorrhage and N-Terminal Pro–Brain Natriuretic Peptide, a Biochemical Marker of LV Remodeling, at 6 Months

Myocardial hemorrhage was associated with N-terminal pro–brain natriuretic peptide at 6 months (regression coefficient, 174.57 [95% confidence interval, 37.85–311.29]; *P*=0.013), after adjustment for baseline LV ejection fraction, baseline LV end-diastolic volume, and baseline N-terminal pro–brain natriuretic peptide (Results section in the Data Supplement).

### Myocardial Hemorrhage, Microvascular Obstruction, and Longer-Term Health Outcomes

Two hundred forty-five (100%) patients had longer term follow-up completed. The median duration of follow-up was 830 days. Ten of 245 (4%) patients had a cardiovascular cause of death or experienced a heart failure event post discharge. Myocardial hemorrhage was associated with cardiovascular death or first hospitalization for heart failure post discharge, (hazard ratio, 5.89; 95% confidence interval, 1.25–27.74; *P*=0.025), including after adjustment for baseline LV end-diastolic volume. Microvascular obstruction (*P*=0.22) and the hypointensive core on T2 mapping (*P*=0.31) were not associated with cardiovascular death or heart failure post discharge.

## Discussion

We have undertaken the largest clinical study to date of myocardial hemorrhage using diagnostic T2* CMR mapping in a comparatively large unselected population of patients with acute STEMI. We have also reported for the first time a serial imaging time-course study of myocardial hemorrhage and microvascular obstruction.

Our main findings are as follows: (1) the incidence of myocardial hemorrhage was 41%, and hemorrhage occurred less frequently and was less extensive in size than microvascular obstruction revealed by contrast-enhanced CMR or the hypointense core revealed by T2 mapping; (2) myocardial hemorrhage was associated with clinical characteristics indicative of more severe MI and systemic inflammation; (3) myocardial hemorrhage was independently associated with adverse LV remodeling at 6 months and cardiovascular death and first hospitalization for heart failure; (4) considering the time course, myocardial hemorrhage occurred in ≈1 quarter of the patients within 12 hours of reperfusion, and the incidence approximately doubled by day 2, indicating secondary progression after the acute bleed; (5) the early progressive evolution of myocardial hemorrhage was distinct from microvascular obstruction that was maximal 4 to 12 hours post MI and then decreased subsequently; (6) a hypointense infarct core on T2 mapping always occurred in the presence of microvascular obstruction and commonly in the absence of myocardial hemorrhage within 12 hours and 2 days post MI, indicating that the presence of T2 core is more closely associated with microvascular obstruction than myocardial hemorrhage; and (7) myocardial hemorrhage was more consistently associated with adverse surrogate outcomes (LV remodeling and N-terminal pro–brain natriuretic peptide) and spontaneous adverse health outcomes (cardiovascular death or heart failure post discharge) than microvascular obstruction.

To date, our understanding of the natural history of reperfusion hemorrhage has been limited by constraints with diagnostic methods. Most studies have used dark blood T2-weighted imaging to detect hemorrhage^10–13,18^; however, this qualitative technique is hampered by imaging artifact,^34^ false-positive effects of microvascular obstruction,^35,36^ and lack of specificity for the detection of myocardial hemorrhage. Methodological issues, such as the use of dark-blood T2-weighted imaging and the inclusion of incident adverse cardiac events during the index hospitalization, may have confounded the results of previous prognostic studies such that the clinical significance of myocardial hemorrhage, as a deleterious complication or simple bystander of infarct severity, was uncertain. Taken together, our results indicate that the time course and prognostic significance of myocardial hemorrhage are distinctly significant.

T2-weighted imaging is strongly influenced by edema, and the hyperintense signal from edema may mask a hypointense signal because of localized infarct core hemorrhage.^22^ On the other hand, T2* imaging is relatively insensitive to the effects of edema.^8^ Quantitative T2 mapping addresses the limitations associated with T2-weighted techniques,^22,34–36^ offers increased accuracy in the detection of myocardial edema, and may provide a more objective assessment of the infarct core because T2 relaxation times are more directly estimated.^19–21^ We observed that the hypointense core on T2 mapping is more closely associated with late microvascular obstruction than myocardial hemorrhage, as specifically revealed by T2* mapping.

To date, the temporal evolution of myocardial hemorrhage and its relationship with microvascular obstruction early after reperfusion have been incompletely understood. Experimental studies have noted that hemorrhage occurs as a consequence of reperfusion,^37^ whereas other studies have observed that hemorrhage may be a secondary phenomenon because of progressive capillary breakdown.^6,7,9^ A recent experimental study by Robbers et al^7^ indicated that microvascular obstruction might be a modifiable precursor of hemorrhage, which represented irreversible microvascular destruction. In our study, we observed that microvascular obstruction occurred maximally 4 to 12 hours post reperfusion, whereas, by contrast, myocardial hemorrhage increased progressively from a zero baseline, through 4 to <12 hours to a peak 2 days after reperfusion followed by a reduction at 10 days. Also, in accordance with other recent studies using T2* imaging,^8,9,16,17,21^ we observed that hemorrhage only occurred within regions of microvascular obstruction. Therefore, in agreement with the preclinical findings of Robbers et al,^7^ our time-course analysis supports the conclusion that hemorrhage is a downstream consequence of microvascular obstruction.

Previous studies using dark-blood T2-weighted imaging to define hemorrhage showed that microvascular obstruction occurred commonly in the absence of a T2 hypointense core.^18^ In contrast, we observed that all patients with microvascular obstruction had a hypointense core on T2 mapping. Our results are supported by a postmortem analysis,^36^ which showed that a T2 hypointense core always represented microvascular obstruction, with or without hemorrhage and by a recent clinical study by Symons et al^13^ in 186 patients with recent STEMI. In their study, all patients with a hypointense core on dark-blood T2-weighted CMR had concomitant microvascular obstruction, and the combination of these pathologies was more strongly associated with adverse LV remodeling than microvascular obstruction alone. Our analysis extends these findings by identifying associations between myocardial hemorrhage revealed by T2* CMR and baseline clinical characteristics (ie, the severity of MI and inflammation) and multivariable associations with LV remodeling and with adverse health outcomes.

We also observed that the presence and extent of a hypointense core disclosed by T2 mapping were more closely related to early microvascular obstruction than late microvascular obstruction or hemorrhage. We postulate that the occurrence of a T2 hypointense core in the absence of hemorrhage most likely represents a reduction in the effective tissue water content within the infarct core because of obstruction of capillary blood flow (eg, cellular debris and extrinsic edema) and microvascular spasm, thereby reducing the amount of tissue water content and shortening T2 relaxation (ms) within the infarct core.^19^ Furthermore, we observed that the size of the hypointense core on T2 imaging was greater than the extent of microvascular obstruction and hemorrhage (Figure 3) consistent with observations in experimental MI.^7^ There are conflicting data on the temporal change in size of microvascular obstruction in the early reperfusion period. Canine studies^38,39^ have shown that the amount of microvascular obstruction increases in the first 48 hours after reperfusion and then remains stable between 2 and 9 days. However, there have not been any confirmatory studies in humans to demonstrate expansion of microvascular obstruction at any time point post reperfusion. Our findings indicate that the extent of microvascular obstruction remains stable between 4 to 12 hours and day 2 and then decreases to day 10, which is in agreement with other clinical data.^40,41^

Our results have important clinical implications. Because hemorrhage increases progressively early and post MI, it may be modifiable with targeted preventive therapy to restore microvascular perfusion and preserve capillary integrity. This possibility is undergoing prospective assessment in T-TIME (NCT02257294), which is a randomized placebo-controlled clinical trial of reduced doses of alteplase for intracoronary thrombolysis immediately after reperfusion during primary PCI. Alternatively, CMR with T2* mapping could be used to identify myocardial hemorrhage in STEMI survivors who might benefit from more intensive therapy or novel therapies designed to prevent adverse LV ventricular remodeling. Further studies are warranted.

### Limitations

Our study lacks pathological correlation of the imaging results. As a result of time constraints reflecting the duration of the CMR scan, we only acquired 3 short-axis slices using T2* mapping, and therefore, minor degrees of hemorrhage could have been missed. The amount of hemorrhage was calculated from 3 slice positions (basal, mid, and apical) rather than a full LV stack to constrain the total duration of the CMR scan. The T2* acquisition was associated with imaging artifacts that limited the quantification of hemorrhage in some patients, and only 86% of the initial cohort had analyzable T2* data. Future improvements to T2* mapping could include the use of high-pass filtered processing^42^ and the use of an automated truncation method.^43^

### Conclusions

We found that myocardial hemorrhage occurs commonly and follows a progressive time course within the first 2 days post MI that is distinct from microvascular obstruction. The occurrence of myocardial hemorrhage is prognostically more significant than microvascular obstruction. Our findings are relevant for risk assessment in STEMI survivors and for the development and assessment of novel therapies to limit reperfusion injury and infarct size post STEMI.

## Acknowledgments

We thank the patients who participated in this study and the staff from the Departments Cardiology and Radiology. We thank Peter Weale and Patrick Revell (Siemens Healthcare, United Kingdom).

## Sources of Funding

This research was supported by the British Heart Foundation (BHF) Grant (Project Grant PG/11/2/28474), the National Health Service, and the Chief Scientist Office. In addition, the research was supported by a nonfinancial research agreement with Siemens Healthcare. C. Berry was supported by a Senior Fellowship from the Scottish Funding Council. Dr Welsh is supported by BHF Fellowship FS/12/62/29889.

## Disclosures

None.

## Supplementary Material

## CLINICAL PERSPECTIVE

Myocardial perfusion remains compromised in ≤50% of patients with ST-segment–elevation myocardial infarction, despite rapid and successful mechanical revascularization of the culprit artery. Although pathological and clinical observations initially seemed to support the theory that no reflow was a consequence of microvascular obstruction, irreversible microvascular injury and subsequent myocardial hemorrhage are now also thought to play important factors in this process. The clinical significance of myocardial hemorrhage is uncertain, as is the temporal evolution and inter-relationships with microvascular obstruction. T2* cardiac magnetic resonance is considered the gold standard for in vivo myocardial hemorrhage visualization post myocardial infarction. This study shows that myocardial hemorrhage is associated with adverse remodeling and adverse health outcome in the longer term. In addition, the occurrence of myocardial hemorrhage is prognostically more significant than microvascular obstruction. The results of our serial imaging time-course substudy show that myocardial hemorrhage follows a progressive time course within the first 2 days post myocardial infarction and is distinct from microvascular obstruction, which decreases with time post reperfusion. Our findings are relevant for risk assessment in ST-segment–elevation myocardial infarction survivors. Furthermore, a detailed knowledge of the early dynamic changes of both hemorrhage and microvascular obstruction is critical to the development and assessment of novel therapies to limit reperfusion injury and infarct size post ST-segment–elevation myocardial infarction.
