# Supplementary material for: Myocardial Hemorrhage After Acute Reperfused ST-Segment–Elevation Myocardial Infarction: Relation to Microvascular Obstruction and Prognostic Significance
Source: Circ Cardiovasc Imaging. 2016 Jan 19;9(1):e004148. doi: 10.1161/CIRCIMAGING.115.004148 (PMC4718183; doi:10.1161/CIRCIMAGING.115.004148)
Supplement: Supplementary file 1 [file hci-9-e004148-s001.pdf]

Supplementary Material

Myocardial hemorrhage after acute reperfused ST-elevation myocardial infarction: relation to microvascular obstruction and prognostic significance.

ClinicalTrials.gov registration NCT02072850

Table of contents

Supplementary Methods 3
Setting and study populations 3
Coronary angiogram acquisition and analyses 4
Percutaneous coronary intervention 4
Angiographic analysis 5
Definitions 5
CMR acquisition and analyses 6
Electrocardiogram 10
Biochemical and hematologic measurement of inflammation 10
Hematologic measurement of inflammation 11
Statistics 12
Supplementary Results 15
Supplementary Figure Legends 37
Supplementary Figures 38
References 44

Supplementary Methods

Setting and study populations

STEMI patients

Screening, enrolment, and data collection were prospectively performed by cardiologists in the cardiac catheterization laboratories of the Golden Jubilee National Hospital, Glasgow, United Kingdom. This hospital is a regional referral centre for primary and rescue percutaneous coronary intervention (PCI). The hospital provides clinical services for a population of 2.2 million. A screening log was recorded, including patients who did not participate in the cohort study.

Healthy volunteers

The purpose of including healthy volunteers was to collect normative reference data for myocardial native T2/ T2* in individuals without prior cardiovascular disease or therapy and who were reasonably representative of the population of individuals from whom the STEMI patients were drawn. Second, the reference native T2/ T2* values were required to be measured on the same CMR scanner and with the same protocol that was used for the STEMI patients including during the same time-period.

Healthy volunteers were invited to participate by placing adverts in public buildings (e.g. hospital, University) and through personal contacts of the researchers. Matching and selection of the healthy volunteers was done by the researchers in order to reflect the age and gender distribution of the STEMI patients. The healthy volunteers were resident in the same catchment area as the STEMI population. Fifty age- and gender-matched healthy volunteers who had a normal ECG and no prior history of cardiovascular disease or therapy underwent CMR during the same time period. The absence of late gadolinium enhancement (myocardial

fibrosis or scar) was determined qualitatively by visual assessment, and the absence of late gadolinium enhancement was a requirement for inclusion of the volunteer in this analysis.

The rationale for including healthy volunteers in this study is as follows. First, native T2/ T2* values may vary between CMR scanners and so a local reference range for native T2/ T2* is recommended in CMR guidelines [1]. Second, native T2/ T2* may vary spatially in the heart and therefore. Myocardial native T2/ T2* values were regionally segmented in regions-of-interest and summarised according to the AHA model [2].

Coronary angiogram acquisition and analyses

Coronary angiograms were acquired during usual care with cardiac catheter laboratory X-ray (Innova®) and IT equipment (Centricity®) made by GE Healthcare. The coronary anatomy and disease characteristics of study participants were described based on the clinical reports of the attending cardiologist.

Percutaneous coronary intervention

Consecutive admissions with acute ST-elevation myocardial infarction (STEM) referred for emergency percutaneous coronary intervention (PCI) were screened for the inclusion and exclusion criteria. During ambulance transfer to the hospital, the patients received 300 mg of aspirin, 600 mg of clopidogrel and 5000 IU of unfractionated heparin [3, 4]. The initial primary PCI procedure was performed using radial artery access. A conventional approach to primary PCI was adopted in line with usual care in our hospital [3, 4]. Conventional bare metal and drug eluting stents were used in line with guideline recommendations and clinical judgement. The standard transcatheter approach for reperfusion involves minimal intervention with aspiration thrombectomy only or minimal balloon angioplasty (e.g. a compliant balloon sized according

to the reference vessel diameter and inflated at 4-6 atmospheres 1-2 times). During PCI, glycoprotein IIbIIIa inhibitor therapy was initiated with high dose tirofiban (25 μg/kg/bolus) followed by an intravenous infusion of 0.15 μg/kg/min for 12 hours, according to clinical judgement and indications for bail-out therapy [3, 4]. No reflow was treated according to contemporary standards of care with intra-coronary nitrate (i.e. 200 μg) and adenosine (i.e. 30 – 60 μg) [3, 4], as clinically appropriate. In patients with multivessel coronary disease, multivessel PCI was not recommended, in line with clinical guidelines [3, 4]. The subsequent management of these patients was symptom-guided.

Angiographic analysis

The coronary anatomy and disease characteristics of study participants were described based on the clinical reports of the attending cardiologist.

Definitions

Coronary blood flow can be described based on the visual assessment of coronary blood flow revealed by contrast injection into the coronary arteries [3, 4].

TIMI Coronary Flow Grade	
0	No flow
1	Minimal flow past obstruction
2	Slow (but complete) filling and slow clearance
3	Normal flow and clearance

CMR acquisition and analyses

CMR acquisition

CMR was performed on a Siemens MAGNETOM Avanto (Erlangen, Germany) 1.5-Tesla scanner with a 12-element phased array cardiac surface coil. T2 maps were acquired in contiguous short axis slices covering the whole ventricle, using an investigational prototype T2-prepared (T2P) TrueFisp sequence [5, 6]. Typical imaging parameters were: bandwidth ~947 Hz/pixel; flip angle 70°; T2 preparations: 0 ms, 24 ms, and 55 ms respectively; matrix 160 x 105 pixels; spatial resolution 2.6 x 2.1 x 8.0 mm; slice thickness 8 mm.

T2*-maps were obtained using an investigational prototype T2* map sequence acquired in 3 short-axis slices (basal, mid and apical). Typical imaging parameters were: bandwidth ~814 (x8) Hz/pixel; flip angle 18°; matrix 256x115; spatial resolution 2.6 x 1.6 x 10 mm; slice thickness 8 mm.

Late gadolinium enhancement images covering the entire LV were acquired 10-15 minutes after intravenous injection of 0.15 mmol/kg of gadoterate meglumine (Gd^{2+}-DOTA, Dotarem, Guebert S.A.) using segmented phase-sensitive inversion recovery (PSIR) turbo fast low-angle shot [7]. Microvascular obstruction was defined as a dark zone on early delayed enhancement imaging 1, 3, 5 and 7 minutes post-contrast injection and within an area of late gadolinium enhancement. Typical imaging parameters were: matrix = 192 x 256, flip angle = 25°, TE = 3.36 ms, bandwidth = 130 Hz/pixel, echo spacing = 8.7ms and trigger pulse = 2. The voxel size was 1.8 x 1.3 x 8 mm^3. Inversion times were individually adjusted to optimize nulling of apparently normal myocardium (typical values, 200 to 300 ms).

MR image analyses

The images were analysed on a Siemens work-station by observers with at least 3 years CMR experience (N.A., D.C., I.M, S.R.). All of the images were reviewed by experienced CMR cardiologists (C.B., N.T.). LV dimensions, volumes and ejection fraction were quantified using computer assisted planimetry (syngo MR®, Siemens Healthcare, Erlangen, Germany). All scan acquisitions were spatially co-registered.

T2 and T2 – standardized measurements in myocardial regions of interest*

LV contours were delineated with computer assisted planimetry on the raw T2* image and the last corresponding T2 raw image, with echo time of 55 ms [8]. Contours were then copied onto the color-encoded spatially co-registered maps and corrected when necessary by consulting the SSFP cine images. Apical segments were not included because of partial volume effects. Particular care was taken to delineate regions of interest with adequate margins of separation from tissue interfaces prone to partial volume averaging such as between myocardium and blood. Each T2/ T2* map image was visually assessed for the presence of artifacts relating to susceptibility effects or cardio-respiratory motion. Each map was evaluated against the original images. When artifacts occurred, the affected segments were not included in the analysis.

T2/ T2* values were segmented spatially and regions of interest were defined as (1) remote myocardium, (2) injured myocardium and (3) infarct core. The regions-of-interest were planimetered to include the entire area of interest with distinct margins of separation from tissue interfaces to exclude partial volume averaging. The remote myocardial region-of-interest was defined as myocardium 180º from the affected zone with no visible evidence of infarction, edema or wall motion abnormalities (assessed by inspecting corresponding contrast enhanced T1-weighted, T2-weighted and cine images, respectively). The infarct zone

region-of-interest was defined as myocardium with pixel values (T2) >2 SD from remote myocardium on T2-weighted CMR [5, 6]. The infarct core was defined as an area in the center of the infarct territory having a mean T2/ T2* value of at least 2 standard deviations (SDs) below the T2/ T2* value of the periphery of the area-at-risk.

In healthy volunteers, the mid-ventricular T2/T2* map was segmented into 6 equal segments, using the anterior right ventricular-LV insertion point as the reference point [2]. T2/T2* was measured in each of these segments, and regions-of-interest were planimetered distinct and separate from blood-pool and tissue interfaces. These segmental values were also averaged to provide one value per subject. Results are presented as average values for segments and slices.

Infarct definition and size

The presence of acute infarction was established based on abnormalities in cine wall motion, rest first-pass myocardial perfusion, and delayed-enhancement imaging. In addition, supporting changes on the ECG and coronary angiogram were also required. Acute infarction was considered present only if late gadolinium enhancement was confirmed on both the axial and long axis acquisitions. The myocardial mass of late gadolinium (grams) was quantified using computer assisted planimetry and the territory of infarction was delineated using a signal intensity threshold of >5 standard deviations above a remote reference region and expressed as a percentage of total LV mass [9]. Infarct regions with evidence of microvascular obstruction were included within the infarct area and the area of microvascular obstruction was assessed separately and also expressed as a percentage of total LV mass.

Myocardial hemorrhage

Myocardial hemorrhage was scored visually. On the T2* maps, a region of reduced signal intensity within the infarcted area, with a T2* value of <20 ms [10-13], was considered to confirm the presence of myocardial hemorrhage.

Area-at-risk

Area-at-risk was defined as LV myocardium with pixel values (T1/T2) >2 standard deviations from remote myocardium [5, 6, 14-17]. In order to assess the area-at-risk the epicardial and endocardial contours on the last corresponding T2-weighted raw image with an echo time of 55 ms were planimetered [8]. Contours were then copied to the map and corrected when necessary by consulting the SSFP cine images.

Myocardial salvage

Myocardial salvage was calculated by subtraction of percent infarct size from percent area-at-risk [14, 17, 18]. The myocardial salvage index was calculated by dividing the myocardial salvage area by the initial area-at-risk.

Adverse remodelling

Adverse remodelling was defined as an increase in LV end-diastolic volume ≥ 20% at 6 months from baseline [19].

Reference ranges

Reference ranges used in the laboratory were 105 – 215 g for LV mass in men, 70 – 170 g for LV mass in women, 77 – 195 ml for LV end-diastolic volume in men, 52 – 141 ml for LV end-diastolic volume in women, 19 – 72 ml for LV end-systolic volume in men and 13 – 51 ml for LV end-systolic volume in women.

Electrocardiogram

A 12 lead electrocardiogram (ECG) was obtained before coronary reperfusion and 60 minutes afterwards with Mac-Lab® technology (GE Healthcare) in the catheter laboratory and a MAC 5500 HD recorder (GE Healthcare) in the Coronary Care Unit. The ECGs were acquired by trained cardiology staff. The ECGs were de-identified and transferred to the local ECG management system. The ECGs were then analysed by the University of Glasgow ECG Core Laboratory which is certified to ISO 9001: 2008 standards as a UKAS Accredited Organization.

The extent of ST-segment resolution on the ECG assessed 60 minutes after reperfusion compared to the baseline ECG before reperfusion[3] was expressed as complete (≥70%), incomplete (30% to < 70%) or none (≤30%). ECG evidence of reperfusion injury was taken as persistence of ST segment elevation resolution post-procedure, and specifically ≤30% ST-segment resolution post-PCI.

Biochemical and hematologic measurement of inflammation

Serial systemic blood sample were obtained immediately after reperfusion in the cardiac catheterization laboratory, and subsequently between 0600 - 0700 hrs each day during the initial in-patient stay in the Coronary Care Unit. C-reactive protein (CRP) was measured in an NHS hospital biochemistry laboratory using a particle enhanced immunoturbimetric assay method (Cobras C501, Roche),) and the manufacturers calibrators and quality control material, as a biochemical measure of inflammation. The high sensitive assay CRP measuring range is 0.1-250 mg/L. The expected CRP values in a healthy adult are < 5 mg/L, and the

reference range in our hospital is 0 - 10 mg/L. A blood sample was routinely obtained in the cardiac catheter laboratory immediately following revascularization and then again at 0700 hrs on the first and second days after admission to hospital.

NT-proBNP, a biochemical measure of LV wall stress, was measured in a research laboratory using an electrochemiluminescence method (e411, Roche) and the manufacturers calibrators and quality control material. The limit of detection is 5 pg/ml. Long-term coefficient of variations of low and high controls are typically <5%, and were all within the manufacturers range.

Hematologic measurement of inflammation

Leucocyte count and leucocyte sub-populations were measured as a hematologic measure of inflammation using sheath flow technology incorporating semi-conductor laser beam, forward and side scattered light (Sysmex XT200i and XT1800i for white blood cell and differential white blood cell counts, respectively). The linearity ranges for white blood cells was 0.00-440.0 x10(9) /L. The following are the normal ranges for full blood count parameters:

	**MALE**	**FEMALE**
WBC x 10^9/L	4.0 - 11.0	4.0 - 11.0
RBC x 10^12/L	4.50 - 6.50	3.80 - 5.80
Hgb g/L	130 – 180	115 - 165
HCT L/L	0.400 - 0.540	0.370 - 0.470
MCV fL	78 – 99	78 - 99

MCH Pg	27.0 - 32.0	27.0 - 32.0
MCHC g/L	310 – 360	310 - 360
PLATELETS x 10^9/L	150 – 400	150 - 400
NEUTROPHILS x 10^9/L	2.5 - 7.5	2.5 - 7.5
LYMPHOCYTES x 10^9/L	1.5 - 4.0	1.5 - 4.0
MONOCYTES x 10^9/L	0.2 - 0.8	0.2 - 0.8
EOSINOPHILS x 10^9/L	0.0 - 0.4	0.0 - 0.4
BASOPHILS x 10^9/L	0.01 - 0.10	0.01 - 0.10

A blood sample was routinely obtained in the cardiac catheter laboratory, immediately following revascularization and then again at 0700 on the first and second days after admission to hospital.

Statistics

Sample size calculation for serial imaging longitudinal study

The sample size of 30 was predetermined based on the incidence of infarct pathology (e.g. myocardial hemorrhage or microvascular obstruction) affecting at least one third of the cohort and quantitative data (e.g. T2 ms, edema area (%)) on 4 occasions in all subjects. In order to detect a minimal clinically significant correlation (r) of 0.6 between two continuous variables then a sample size of 25 would be associated with 90% power and 5% significance (null hypothesis, r=0).The sample size aligns with the only other longitudinal CMR study in STEMI survivors with CMR performed on at least 3 occasions [20], and based on what might be feasible within the 3-year duration of this project.

Sample size calculation for the whole cohort

With an estimated hemorrhage incidence of 33% at 48 h post-STEMI, 100 subjects would have evidence of myocardial hemorrhage and 200 subjects would not. The study would have 90% power at a 5% level of significance using a two sided two sample t-test to detect a between-group difference in mean LV end-systolic volume index of 4.65 ml/m2 equivalent to three eighths of a common standard deviation (or an effect size of 0.375). We predicted a between-group difference in mean LVESVI of 4.65 ml/m2 equivalent to three eighths of a common standard deviation (or an effect size of 0.375). We also estimated that at least 30 major adverse cardiac events (MACE) would occur based on a conservative estimate of the event rate (10-12%) at 18 months. The sample size calculation was performed using nQuery version 7.0.

Statistical analysis

Categorical variables are expressed as number and percentage of patients. Most continuous variables followed a normal distribution and are therefore presented as means together with standard deviation. Those variables that did not follow a normal distribution are presented as medians with interquartile range. Differences between groups were assessed using one-way ANOVA, Kruskal-Wallis test or Fisher's where appropriate. Agreement was assessed with Bland Altman plots. Changes over time were assessed using generalized linear mixed effects models (LMEs) with time and haemorrhage as fixed effects, and subject ID as the only random effect. These models were fitted as full factorial models, with the interaction being removed only when not significant or not appropriate. Post-hoc multiple comparisons were performed with Tukey adjustment. Random effects models were used to compute inter-and intra- rater reliability measures (intra-class correlation coefficient (ICC)) for the reliability of remote zone, infarct zone and infarct core T2 and T2* values measured independently by 2 observers in 20 randomly selected patients from the cohort. All statistical analyses were

carried out using R v 2.15.1 or SAS v9.3, or later versions of these programs. A p-value > 0.05 indicates the absence of evidence for a statistically significant effect.

Trial Management

The study was conducted in line with Guidelines for Good Clinical Practice (GCP) in Clinical Trials. http://www.mrc.ac.uk/documents/pdf/good-clinical-practice-in-clinical-trials/

Trial management included a Trial Management Group, and an independent Clinical Trials Unit. Day to day study activity was coordinated by the Trial Management Group who was responsible to the Sponsor which was responsible for overall governance and that the trial was conducted according to GCP standards.

Clinical events were assessed and validated by an independent cardiologist (A.M.) who had access to relevant source clinical data. This cardiologist followed an agreed charter and he was blinded to all of the other clinical data.

Supplementary Results

Supplementary Table 1. Association of patient characteristics with myocardial hemorrhage, as defined by T2* CMR, in multivariable stepwise regression analyses (n=245).

Multiple stepwise regression	Odds ratio (95% CI)	p value
A. Including patient characteristics and angiographic data		
Male	2.36 (1.15, 4.85)	0.019
Previous PCI	5.92 (1.23, 28.56)	0.027
Smoker	2.45 (1.21, 4.96)	0.013
Killip class >2	15.13 (1.86, 123.12)	0.011
TIMI flow >1 at initial angiography	0.27 (0.13, 0.56)	<0.001
≤30% ST-segment resolution post-PCI	3.08 (1.27, 7.50)	0.013
B. Including patient characteristics, angiographic data, and LVEF		
LVEF	0.93 (0.89, 0.97)	<0.001

Male	2.25 (1.08, 4.71)	0.030
Previous PCI	5.52 (1.22, 25.10)	0.027
Smoker	2.44 (1.19, 5.00)	0.015
TIMI flow >1 at initial angiography	0.27 (0.13, 0.57)	<0.001
C. Including patient characteristics, angiographic data, and LVEDV		
LVEDV	1.02 (1.01, 1.03)	<0.001
Previous PCI	6.42 (1.38, 29.86)	0.018
Smoker	2.36 (1.15, 4.83)	0.019
Killip class >2	10.62 (1.27, 89.02)	0.029
TIMI flow >1 at initial angiography	0.26 (0.12, 0.56)	<0.001
≤30% ST-segment resolution post-PCI	2.88 (1.17, 7.09)	0.027
D. Including patient characteristics, angiographic data, and peak neutrophil count		
Peak neutrophil count, x10^9/L	1.24 (1.13, 1.37)	<0.001
Male	2.02 (0.99, 4.13)	0.054

Diabetes	2.21 (0.92, 5.77)	0.073
Killip class >2	24.33 (2.98, 198.84)	0.003
D. Including patient characteristics, angiographic data, and peak monocyte count		
Peak monocyte count, x10^9/L	2.95 (1.14, 7.59)	0.025
Male	2.15 (1.04, 4.45)	0.039
Previous PCI	6.15 (1.26, 29.91)	0.024
Smoker	2.19 (1.07, 4.49)	0.033
Killip class >2	14.35 (1.76, 117.13)	0.013
TIMI flow >1 at initial angiography	0.26 (0.12, 0.56)	<0.001
≤30% ST-segment resolution post-PCI	2.84 (1.14, 7.06)	0.025

The clinical and angiographic characteristics that were assessed are listed in Table 1. Separate multivariable analyses were performed for (A) patient characteristics and angiographic data and (B) CMR data. CMR parameters, which were all highly correlated with one another, were included separately in multiple stepwise regression models with patient characteristics and angiographic data to reduce multicollinearity.

Supplementary Table 2. Clinical and angiographic characteristics of the 30 patients in the longitudinal clinical study.

Characteristics*	All Patients<br>n = 30	No Haemorrhage or MVO<br>n = 14	MVO only<br>n = 3	Hemorrhage and MVO<br>n = 13	p value
Clinical					
Age, years	54 (10)	56 (10)	52 (10)	53 (11)	0.649
Male sex, n (%)	25 (83)	12 (86)	3 (100)	10 (77)	0.801
BMI, (kg/m^2)	28 (5)	30 (5)	28 (1)	27 (5)	0.608
History					
Hypertension, n (%)	8 (27)	5 (36)	0 (0)	3 (21)	0.598
Current smoking, n (%)	21 (70)	10 (71)	1 (33)	10 (77)	0.442
Hypercholesterolemia, n (%)	13 (43)	7 (50)	0 (0)	6 (46)	0.376
Diabetes mellitus‡, n (%)	2 (7)	1 (7)	0 (0)	1 (8)	1.000
Previous angina, n (%)	3 (10)	1 (7)	0 (0)	2 (15)	0.709

Previous myocardial infarction, n (%)		1 (3)	1 (7)	0 (0)	0 (0)	1.000
Previous PCI, n (%)		30 (100)	14 (100)	3 (100)	13 (100)	-
Presenting characteristics						
Heart rate, bpm		77 (17)	78 (20)	60 (6)	80 (14)	0.084
Systolic blood pressure, mmHg		141 (26)	147 (31)	102 (2)	143 (16)	0.020
Diastolic blood pressure, mmHg		84 (12)	86 (12)	66 (2)	86 (11)	0.026
Time from symptom onset to reperfusion, min		155 (112, 243)	159 (101, 174)	300 (212, 314)	136 (112, 274)	0.473
Ventricular fibrillation†, n (%)		1 (3)	1 (7)	0 (0)	0 (0)	1.000
Heart failure, Killip class at presentation, n (%)	I	22 (73)	12 (86)	1 (33)	9 (69)	
	II	7 (23)	2 (14)	2 (67)	3 (23)	0.219
	III/IV	1 (3)	0 (0)	0	1 (8)	
ECG						
ST segment elevation resolution post PCI, n (%)						
None, ≤30%		2 (7)	1 (7)	0 (0)	1 (8)	1.000

Partial, 30% to < 70%		13 (43)	6 (42)	1 (33)	6 (46)	
Complete, ≥70 %		15 (50)	7 (50)	2 (67)	6 (46)	
Coronary angiography						
Reperfusion strategy, n (%)						
Primary PCI		30 (100)	14 (100)	3 (100)	13 (100)	
Rescue PCI (failed thrombolysis)		0 (0)	0 (0)	0 (0)	0 (0)	1.000
Successful thrombolysis		0 (0)	0 (0)	0	0 (0)	
Number of diseased arteries¥, n (%)	1	14 (47)	8 (57)	2 (67)	4 (31)	
	2	11 (37)	4 (29)	1 (33)	6 (46)	0.674
	3	5 (17)	2 (14)	0 (0)	3 (23)	
	LM	0 (0)	0 (0)	0 (0)	0 (0)	
Culprit artery, n (%)	LAD	9 (30)	4 (29)	0 (34)	5 (38)	
	LCX	10 (33)	3 (21)	1 (33)	6 (46)	0.234
	RCA	11 (37)	7 (50)	2 (67)	2 (15)	

TIMI coronary flow grade pre-PCI, n (%)	0/1	24 (80)	9 (64)	3 (100)	12 (92)	0.192
	2/3	6 (20)	5 (36)	0 (0)	1 (8)	
TIMI coronary flow grade post-PCI, n (%)	0/1	0 (0)	0 (0)	0	0 (0)	1.000
	2/3	30 (100)	14 (100)	3 (100)	13 (100)	
Initial blood results on admission						
C-reactive protein, (mg/L)		3 (1, 6)	4 (1, 6)	1 (1, 34)	2 (1, 5)	0.667
Leucocyte cell count (x10^{9}L)		13.2 (3.4)	12.8 (3.5)	10.7 (1.8)	14.2 (3.4)	0.263
Neutrophil count (x10^{9}L)		10.2 (3.1)	9.6 (3.1)	7.8 (1.5)	11.3 (3.0)	0.141
Monocytes (x10^{9}L)		0.9 (0.3)	0.9 (0.3)	0.9 (0.3)	1.0 (0.4)	0.887
NT-proBNP, pg/mL		767 (369, 1633)	566 (260, 1412)	799 (432, 1946)	1044 (646, 1642)	0.295
Medication at discharge						
ACE-inhibitor		30 (100)	14 (100)	3 (100)	13 (100)	1.000
Beta-blocker		30 (100)	14 (100)	3 (100)	13 (100)	1.000

*P-values were obtained from t-tests or Mann-Whitney test as appropriate for continuous variables, and Fisher’s tests for categorical variables.

Table 3. CMR findings of serial imaging subset (n=30) at four time-points post-reperfusion.

	4 < 12 hours<br>n = 30	3 Days<br>n = 30	10 Days<br>n =30	6-7 months<br>n =30
LV ejection fraction, %	52 (9)	56 (9)	59 (8)	59 (8)
LV end-diastolic volume, ml	158 (135, 184)	165 (129, 181)	164 (132, 194)	161 (120, 196)
Area at risk, % LV mass	34 (10)	39 (12)	31 (12)	-
Infarct size, % LV mass	19 (13)	20 (13)	14 (10)	14 (10)
Late microvascular obstruction present, n (%)	18	17	10	0
Early microvascular obstruction, n (%)	20	17	15	-
T2 hypointense core present, n (%)	19	18	14	-
Myocardial hemorrhage present, n (%)	7	13	11	4

Supplementary Table 4. CMR findings in STEMI survivors who participated in the serial imaging sub-study. The results are presented for scans obtained 3 days and 6 months post-MI in 28 STEMI patients with complete data for myocardial hemorrhage and microvascular obstruction (MVO).*

Characteristics*	All patients	No Hemorrhage and no MVO	MVO only	Hemorrhage and MVO	p-value
	n = 30	n = 14	n = 3	n = 13	
CMR findings 3 days post-MI					
LV ejection fraction, %	55 (9)	57 (8)	62 (14)	52 (8)	0.217
LV end-diastolic volume, ml					
Men	170 (33)	166 (38)	176 (16)	173 (31)	0.829
Women	130 (18)	142 (27)	-	123 (6)	0.564
LV end-systolic volume, ml					
Men	77 (26)	75 (30)	68 (29)	82 (21)	0.492
Women	60 (14)	60 (27)	-	60 (6)	1.000

LV mass, g					
Men	148 (22)	147 (17)	142 (36)	152 (26)	0.867
Women	104 (20)	112 (35)	-	99 (10)	1.000
Edema and infarct characteristics					
Area at risk, % LV mass	33 (11)	27 (10)	30 (11)	40 (8)	0.007
Infarct size, % LV mass	17 (12)	8 (5)	17 (5)	27 (10)	<0.001
Myocardial salvage, % of LV mass*	19 (9)	21 (9)	17 (12)	18 (8)	0.661
Myocardial salvage index, %*	60 (23)	78 (14)	51 (23)	45 (19)	<0.001
Late microvascular obstruction present, n (%)	16 (53)	0	3 (100)	13 (100)	<0.001
Late microvascular obstruction, % LV mass	1 (0, 6)	0	3 (2, 4)	7 (3, 9)	<0.001
CMR findings 6 months post-MI (n = 30)					
LV ejection fraction, %	61 (9)	65 (8))	65 (8)	57 (9)	0.064
Change in LV ejection fraction at 6 months from baseline, %	6 (10)	8 (12)	3 (12)	5 (5)	0.692

LV end-diastolic volume, ml					
Men	170 (32)	154 (29)	168 (23)	189 (30)	0.030
Women	127 (7)	120 (1)	-	132 (4)	0.083
Change in LV end-diastolic volume at 6 months from baseline, ml					
Men	-1 (24)	-12 (22)	-8 (11)	16 (20)	0.011
Women	-3 (23)	-22 (28)	-	9 (9)	0.083
LV end-systolic volume, ml					
Men	66 (26)	55 (20)	59 (17)	82 (27)	0.047
Women	52 (9)	44 (2)	-	57 (7)	0.083
Change in LV end-systolic volume at 6 months from baseline, ml					
Men	-10 (24)	-20 (29)	-9 (20)	0.2 (15)	0.053
Women	-8 (15)	-16 (26)	-	-3 (2)	1.000
LV mass, g					

Men	127 (25)	128 (28)	117 (5)	128 (26)	0.590
Women	88 (10)	90 (16)	-	87 (8)	0.767

Of 120 scans performed in 30 STEMI survivors, 102 (85%) and 117 (98%) CMR scans had evaluable T2- and T2-maps, respectively. 28 patients had complete imaging data for myocardial hemorrhage and microvascular obstruction.

Supplementary Table 5. The temporal evolution of amount (% LV mass) of microvascular obstruction and T2 hypo-intense core in patients with myocardial hemorrhage (n=13), within the first 10 days post-reperfusion.

	4 < 12 hours	3 Days	10 Days	P-value
Late microvascular obstruction, % LV mass	5.3 (2.4, 7.8)	5.4 (2.8, 7.2)	1.3 (0.2, 4.0)	<0.001
T2 hypo-intense core, % LV mass	6.0 (4.7, 7.8)	9.9 (7.2, 10.9)	3.4 (1.6, 5.8)	<0.001
Myocardial hemorrhage, %LV mass	2.7 (0.0, 5.6)	7.0 (4.9, 7.5)	4.1 (2.6, 5.5)	<0.001

Footnote: Amount of microvascular obstruction and T2 hypo-intense core were calculated using full LV coverage, whereas myocardial hemorrhage is derived from 3 scans from the basal, mid and apical slice acquisitions. P-values were obtained from linear mixed effects model with subject as a random factor.

Supplementary Table 6. Multivariable predictors of adverse LV remodelling at 6 months post-STEMI.

Multiple stepwise regression	Odds ratio (95% CI)	p value
*Patient characteristics and angiographic findings**		
Myocardial hemorrhage	2.60 (1.16, 5.86)	0.021
Patient characteristics, angiographic findings and LV end-diastolic volume		
Myocardial hemorrhage	2.64 (1.07, 6.49)	0.035
Killip class 2 heart failure	2.62 (1.04, 6.62)	0.041
LV end-diastolic volume at baseline, ml	0.99 (0.97, 1.00)	0.043

The clinical and angiographic characteristics that were assessed are listed in Table 1. Separate multivariable analyses were performed for (A) patient characteristics and angiographic data and (B) CMR data. CMR parameters, which were all highly correlated with one another, were included separately in multiple stepwise regression models with patient characteristics and angiographic data to reduce multicollinearity.

Supplementary Table 7. Clinical and angiographic characteristics of the patients that were eligible but were not included due to non-evaluable T2* maps.

Characteristics*	All Patients n = 324	Patients who underwent CMR n = 300	Patients with evaluable T2* map n = 245	Patients who did not undergo CMR n = 24	Patients with non-evaluable T2* map n = 55
Clinical					
Age, years	59 (12)	59 (11)	58 (11)	58 (12)	64 (11)
Male sex, n (%)	237 (73)	221 (74)	187 (76)	16 (67)	34 (62)
BMI, (kg/m^2)	29 (5)	29 (5)	28 (5)	29 (5)	32 (5)
History					
Hypertension, n (%)	105 (32)	100 (33)	77 (31)	5 (21)	23 (42)
Current smoking, n (%)	196 (61)	184 (61)	153 (62)	8 (33)	31 (56)
Hypercholesterolemia, n (%)	94 (29)	86 (29)	68 (28)	8 (33)	18 (33)

Diabetes mellitus‡, n (%)		34 (11)	33 (11)	28 (11)	1 (4)	5 (9)
Previous angina, n (%)		40 (12)	37 (12)	31 (13)	3 (13)	6 (11)
Previous myocardial infarction, n (%)		25 (8)	23 (8)	17 (7)	2 (8)	6 (11)
Previous PCI, n (%)		18 (6)	17 (6)	13 (5)	1 (4)	4 (7)
Presenting characteristics						
Heart rate, bpm		78 (17)	78 (17)	78 (16)	71 (16)	79 (19)
Systolic blood pressure, mmHg		135 (25)	136 (25)	136 (25)	131 (24)	134 (24)
Diastolic blood pressure, mmHg		79 (14)	79 (14)	80 (14)	78 (14)	76 (15)
Time from symptom onset to reperfusion, min		174 (120, 315)	176 (122, 318)	176 (123, 324)	150 (96, 284)	173 (120, 265)
Ventricular fibrillation†, n (%)		21 (7)	21 (7)	15 (6)	0 (0)	6 (11)
Heart failure, Killip class at presentation, n (%)	I	233 (72)	213 (71	171 (70)	20 (83)	42 (76)
	II	68 (21)	64 (21)	57 (23)	14 (17)	7 (13)
	III/IV	23 (7)	23 (8)	17 (7)	0 (0)	6 (11)

ECG						
ST segment elevation resolution post PCI, n (%)						
None, ≤30%		48 (15)	46 (15)	38(16)	2 (8)	8 (16)
Partial, 30% to < 70%		127 (39)	119 (40)	99 (41)	8 (33)	20 (36)
Complete, ≥70 %		148 (46)	134 (45)	107 (44)	14 (58)	27 (49)
Coronary angiography						
Reperfusion strategy, n (%)						
Primary PCI		302 (93)	278 (93)	229 (94)	10 (83)	49 (89)
Rescue PCI (failed thrombolysis)		14 (4)	14 (5)	10 (4)	1 (8)	4 (7)
Successful thrombolysis		8 (3)	8 (3)	6 (2)	1 (8)	2 (4)
Number of diseased arteries¥, n (%)	1	174 (54)	162 (54)	132 (54)	12 (50)	30 (55)
	2	99 (31)	88 (29)	70 (29)	11 (46)	18 (33)
	3	45 (14)	44 (15)	37 (15)	1 (4)	7 (13)

	LM	6 (2)	6 (2)	6 (2)	0 (0)	0 (0)
Culprit artery, n (%)	LAD	121 (37)	114 (38)	96 (39)	7 (29)	18 (33)
	LCX	59 (18)	54 (18)	48 (20)	5 (21)	6 (11)
	RCA	144 (44)	132 (44)	101 (41)	12 (50)	31 (56)
TIMI coronary flow grade pre-PCI, n (%)	0/1	236 (73)	218 (73)	180 (74)	18 (75)	38 (69)
	2/3	88 (27)	82 (27)	65 (26)	6 (25)	17 (31)
TIMI coronary flow grade post-PCI, n (%)	0/1	4 (1)	3 (1)	3 (1)	1 (4)	0 (0)
	2/3	320 (99)	297 (99)	242 (99)	23 (96)	55 (100)
Initial blood results on admission						
C-reactive protein, (mg/L)		4 (2, 7)	3 (2, 7)	3 (2, 7)	4 (3, 7)	4 (2,9)
Leucocyte cell count ($x10^9L$)		12.4 (3.5)	12.4 (3.5)	12.5 (3.6)	12.5 (3.7)	11.9 (3.2)
Neutrophil count ($x10^9L$)		9.6 (3.3)	9.6 (3.2)	9.7 (3.3)	9.6 (3.7)	9.1 (2.9)
Monocytes ($x10^9L$)		0.9 (0.4)	0.9 (0.4)	0.9 (0.4)	0.9 (0.3)	0.8 (0.3)

NT-proBNP, pg/mL	864 (345, 1637)	784 (345, 1641)	767 (139, 1633)	1041 (688, 1193)	1096 (325, 2068)
Medication at discharge					
ACE-inhibitor, n (%)	320 (99)	297 (99)	242 (99)	23 (96)	55 (100)
Beta-blocker, n (%)	308 (95)	289 (96)	238 (97	19 (79)	51 (93)

P-values are not presented for these post-hoc analyses

Myocardial hemorrhage and N-terminal pro-brain natriuretic peptide, a biochemical marker of LV remodeling, at 6 months

Blood samples had been collected in STEMI patients enrolled during office hours and N-terminal pro-brain natriuretic peptide (NT-proBNP) results were available in 113 (46%) of 245 patients overall at baseline, and 127 (52%) patients at follow-up. Patients with myocardial hemorrhage had significantly higher NT-proBNP results at 6 month follow-up, compared to patients without evidence of hemorrhage (247 (158, 570) vs. 108 (61, 226) pg/mL; p<0.001).

Myocardial hemorrhage was independently associated with NT-proBNP at 6 months (regression coefficient 174.57 (95% CI 37.85, 311.29); p=0.013), after adjustment for baseline LV ejection fraction, baseline LV end-diastolic volume and baseline NT-proBNP (Supplementary Results).

Intra- and inter-observer agreement of T2 and T2* measurements

T2* values in regions-of-interest in remote zones, injured zones and infarct core, in a subgroup of 20 randomly chosen patients were independently measured by two observers. The intra-class correlation coefficients for reliability of remote T2*, infarct zone T2* and infarct core T2* were 0.69 (95% confidence interval (CI): 0.37, 0.87), 0.75 (0.47, 0.89) and 0.90 (0.77, 0.96); all p<0.001, respectively. Bland-Altman plots showed no evidence of bias (Figures 1-3).

In addition, T2 values in regions-of-interest in remote zones, injured zones and infarct core, in a subgroup of 20 randomly chosen patients were also independently measured by two observers. The intra-class correlation coefficients for reliability of remote T2, infarct zone T2 and infarct core T2 were 0.93 (95% confidence interval (CI): 0.82, 0.97), 0.89 (0.74, 0.95)

and 0.86 (0.68, 0.94); all $p<0.001$, respectively. Bland-Altman plots showed no evidence of bias (Figures 4-6).

T2* and T2 values in healthy volunteers compared to STEMI patients

In healthy subjects, mid-ventricular T2 values were lower in males than females (48.5 (2.1) ms vs. 50.6 (2.5) ms; p=0.003). Overall, the inferior segment had the highest T2 value compared to the anterior segment (50.0 (2.8) ms vs. 49.1 (3.0), respectively; p=0.031). At the mid-ventricular level, mean remote zone native T2 was similar in STEMI patients (49.7 (2.1) ms) and healthy volunteers (49.5 (2.5) ms; p=0.511).

The coefficients of variation CoV for native T2 in the mid-ventricular level with regions-of-interest within myocardial regions were: anterior segment CoV = 6.00; antero-lateral segment CoV = 6.49; antero-septal segment CoV = 6.25; inferior segment CoV = 5.50; infero-lateral segment CoV = 4.58; infero-septal segment CoV = 5.36.

T2* values were similar in healthy volunteers, irrespective of gender or location of measurement. At the mid-ventricular level, mean remote zone T2* values were similar in STEMI patients (31.5 (2.4) ms) and healthy volunteers (31.0 (2.1) ms; p=0.162).

Supplementary Figure Legends

Figure 1. Bland-Altman plot for inter-observer agreement of myocardial remote zone T2* values.

Figure 2. Bland-Altman plot for inter-observer agreement of myocardial injury zone T2* values.

Figure 3. Bland-Altman plot for inter-observer agreement of infarct core T2* values.

Figure 4. Bland-Altman plot for inter-observer agreement of myocardial remote zone T2 values.

Figure 5. Bland-Altman plot for inter-observer agreement of myocardial injury zone T2 values.

Figure 6. Bland-Altman plot for inter-observer agreement of infarct core T2 values.

Supplementary Figures

Figure 1.

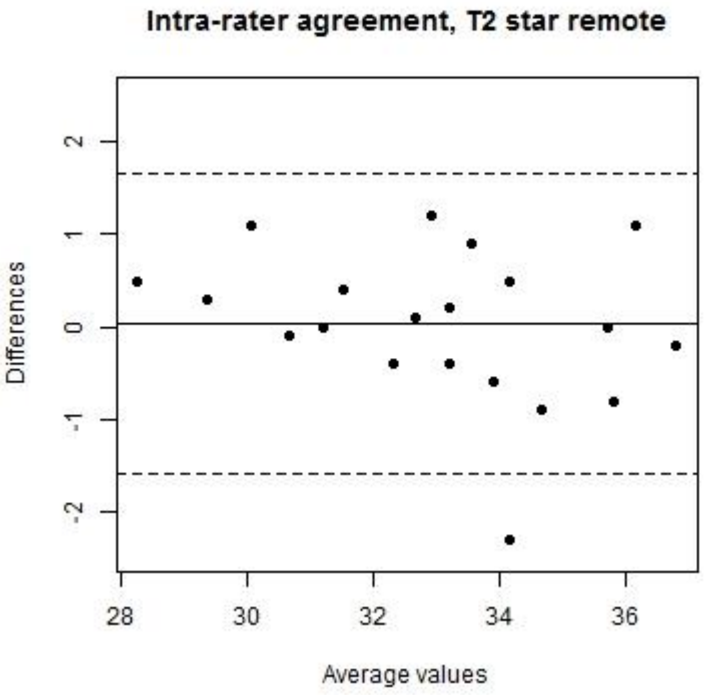

Figure 2.

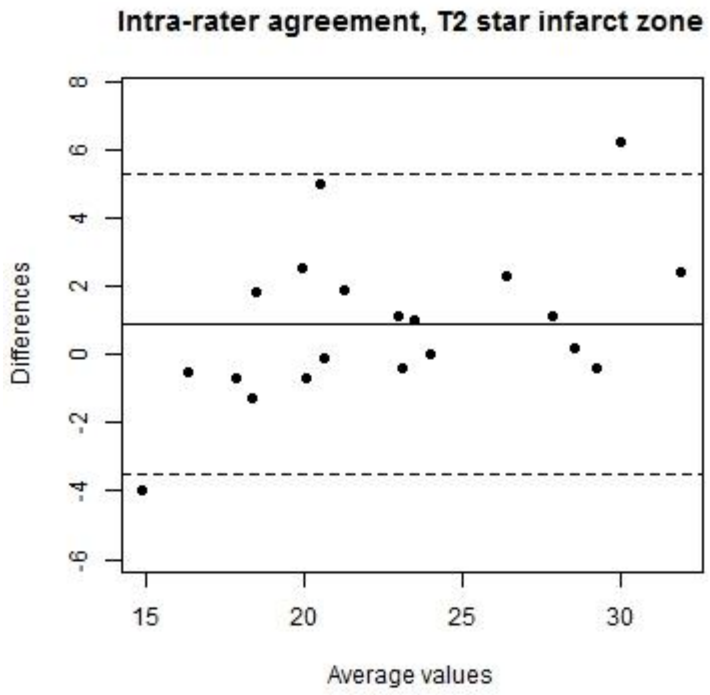
Intra-rater agreement, T2 star infarct zone
Differences
Average values
8
6
4
2
0
-2
-4
-6
15
20
25
30

Figure 3.

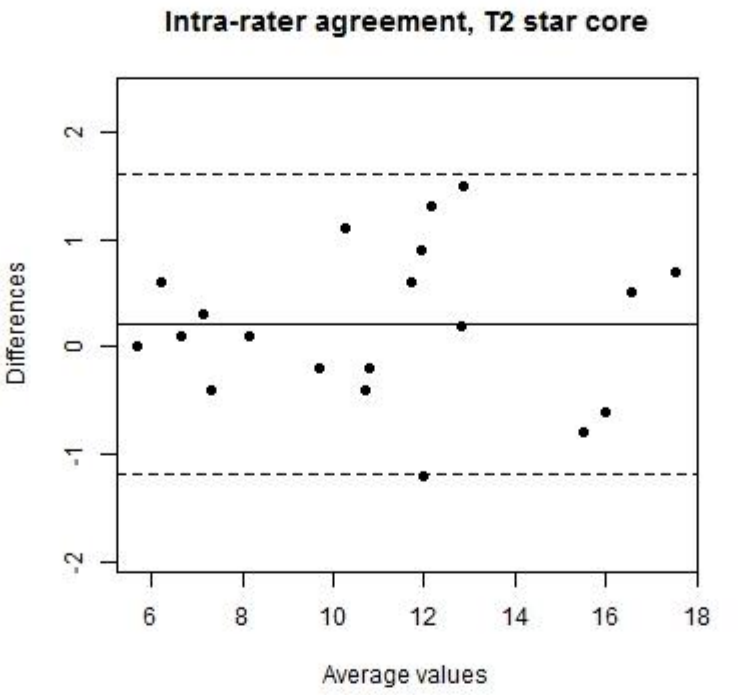

Intra-rater agreement, T2 star core
Differences
2
1
0
-1
-2
6
8
10
12
14
16
18
Average values

Figure 4.

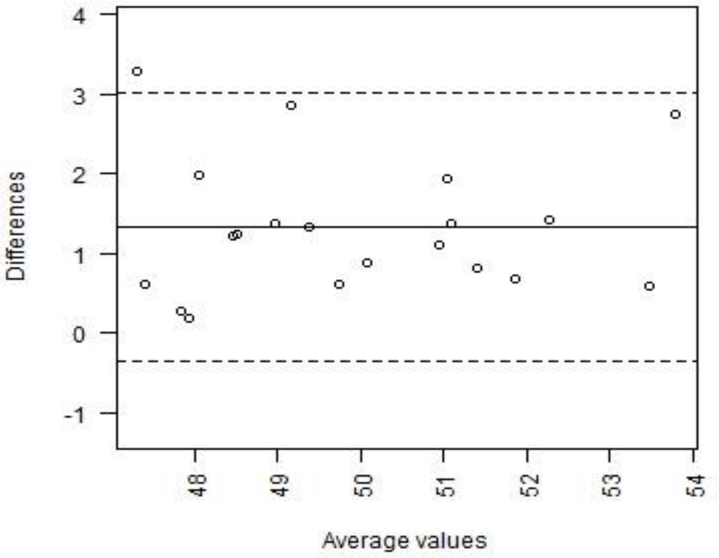

Figure 5.

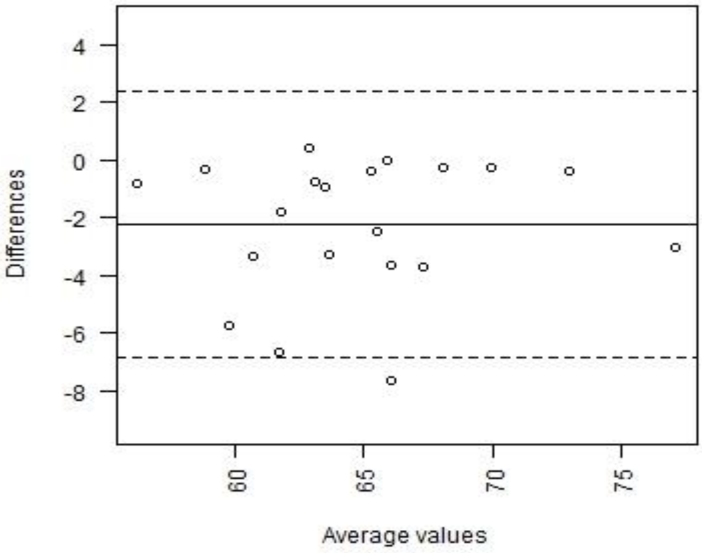

Figure 6.

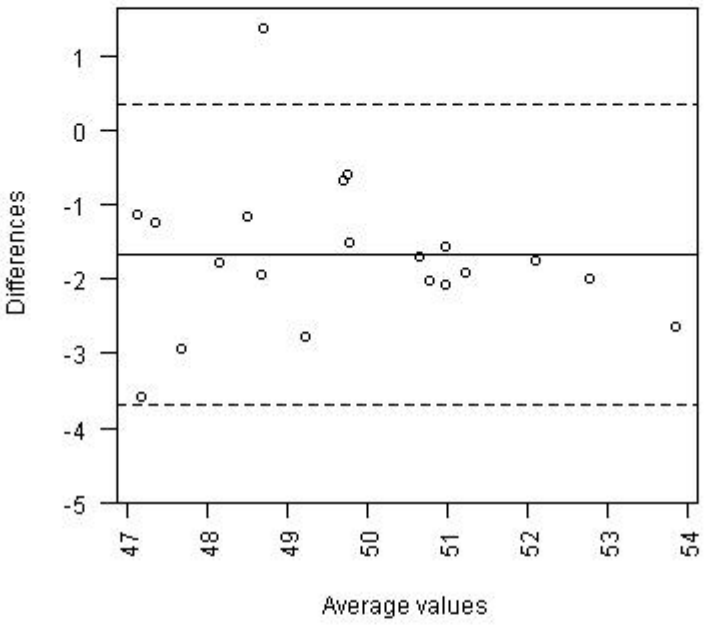

References

1. Kramer CM, Barkhausen J, Flamm SD, Kim RJ and Nagel E. Standardized cardiovascular magnetic resonance (CMR) protocols 2013 update. *Journal of cardiovascular magnetic resonance : official journal of the Society for Cardiovascular Magnetic Resonance*. 2013;15:91.

2. Cerqueira MD, Weissman NJ, Dilsizian V, Jacobs AK, Kaul S, Laskey WK, Pennell DJ, Rumberger JA, Ryan T and Verani MS. Standardized myocardial segmentation and nomenclature for tomographic imaging of the heart. A statement for healthcare professionals from the Cardiac Imaging Committee of the Council on Clinical Cardiology of the American Heart Association. *Circulation*. 2002;105:539-42.

3. Steg PG, James SK, Atar D, Badano LP, Blomstrom-Lundqvist C, Borger MA, Di Mario C, Dickstein K, Ducrocq G, Fernandez-Aviles F, Gershlick AH, Giannuzzi P, Halvorsen S, Huber K, Juni P, Kastrati A, Knuuti J, Lenzen MJ, Mahaffey KW, Valgimigli M, van 't Hof A, Widimsky P and Zahger D. ESC Guidelines for the management of acute myocardial infarction in patients presenting with ST-segment elevation. *European heart journal*. 2012;33:2569-619.

4. Windecker S, Kolh P, Alfonso F, Collet JP, Cremer J, Falk V, Filippatos G, Hamm C, Head SJ, Juni P, Kappetein AP, Kastrati A, Knuuti J, Landmesser U, Laufer G, Neumann FJ, Richter DJ, Schauerte P, Sousa Uva M, Stefanini GG, Taggart DP, Torracca L, Valgimigli M, Wijns W and Witkowski A. 2014 ESC/EACTS Guidelines on myocardial revascularization: The Task Force on Myocardial Revascularization of the European Society of Cardiology (ESC) and the European Association for Cardio-Thoracic Surgery (EACTS)Developed with the special contribution of the European Association of Percutaneous Cardiovascular Interventions (EAPCI). *European heart journal*. 2014;35:2541-619.

5. Giri S, Chung YC, Merchant A, Mihai G, Rajagopalan S, Raman SV and Simonetti OP. T2 quantification for improved detection of myocardial edema. *Journal of cardiovascular magnetic resonance : official journal of the Society for Cardiovascular Magnetic Resonance*. 2009;11:56.

6. Verhaert D, Thavendiranathan P, Giri S, Mihai G, Rajagopalan S, Simonetti OP and Raman SV. Direct T2 quantification of myocardial edema in acute ischemic injury. *JACC Cardiovascular imaging*. 2011;4:269-78.

7. Kellman P, Arai AE, McVeigh ER and Aletras AH. Phase-sensitive inversion recovery for detecting myocardial infarction using gadolinium-delayed hyperenhancement. *Magnetic resonance in medicine : official journal of the Society of Magnetic Resonance in Medicine / Society of Magnetic Resonance in Medicine*. 2002;47:372-83.

8. Wassmuth R, Prothmann M, Utz W, Dieringer M, von Knobelsdorff-Brenkenhoff F, Greiser A and Schulz-Menger J. Variability and homogeneity of cardiovascular magnetic resonance myocardial T2-mapping in volunteers compared to patients with edema. *Journal of cardiovascular magnetic resonance : official journal of the Society for Cardiovascular Magnetic Resonance*. 2013;15:27.

9. Flett AS, Hasleton J, Cook C, Hausenloy D, Quarta G, Ariti C, Muthurangu V and Moon JC. Evaluation of techniques for the quantification of myocardial scar of differing etiology using cardiac magnetic resonance. *JACC Cardiovascular imaging*. 2011;4:150-6.

10. Ghugre NR, Ramanan V, Pop M, Yang Y, Barry J, Qiang B, Connelly KA, Dick AJ and Wright GA. Quantitative tracking of edema, hemorrhage, and microvascular obstruction in subacute myocardial infarction in a porcine model by MRI. *Magnetic resonance in medicine : official journal of the Society of Magnetic Resonance in Medicine / Society of Magnetic Resonance in Medicine*. 2011;66:1129-41.

11. Kandler D, Lucke C, Grothoff M, Andres C, Lehmkuhl L, Nitzsche S, Riese F, Mende M, de Waha S, Desch S, Lurz P, Eitel I and Gutberlet M. The relation between hypointense core, microvascular obstruction and intramyocardial haemorrhage in acute reperfused myocardial infarction assessed by cardiac magnetic resonance imaging. *European radiology*. 2014; 24:3277-88.

12. O'Regan DP, Ariff B, Neuwirth C, Tan Y, Durighel G and Cook SA. Assessment of severe reperfusion injury with T2* cardiac MRI in patients with acute myocardial infarction. *Heart*. 2010;96:1885-91.

13. Anderson LJ, Holden S, Davis B, Prescott E, Charrier CC, Bunce NH, Firmin DN, Wonke B, Porter J, Walker JM and Pennell DJ. Cardiovascular T2-star (T2*) magnetic resonance for the early diagnosis of myocardial iron overload. *European heart journal*. 2001;22:2171-9.

14. Eitel I, Desch S, Fuernau G, Hildebrand L, Gutberlet M, Schuler G and Thiele H. Prognostic significance and determinants of myocardial salvage assessed by cardiovascular magnetic resonance in acute reperfused myocardial infarction. *Journal of the American College of Cardiology*. 2010;55:2470-9.

15. Berry C, Kellman P, Mancini C, Chen MY, Bandettini WP, Lowrey T, Hsu LY, Aletras AH and Arai AE. Magnetic resonance imaging delineates the ischemic area at risk and myocardial salvage in patients with acute myocardial infarction. *Circulation Cardiovascular imaging*. 2010;3:527-35.

16. Payne AR, Casey M, McClure J, McGeoch R, Murphy A, Woodward R, Saul A, Bi X, Zuehlsdorff S, Oldroyd KG, Tzemos N and Berry C. Bright-blood T2-weighted MRI has higher diagnostic accuracy than dark-blood short tau inversion recovery MRI for detection of acute myocardial infarction and for assessment of the ischemic area at risk and myocardial salvage. *Circulation Cardiovascular imaging*. 2011;4:210-9.

17. Francone M, Bucciarelli-Ducci C, Carbone I, Canali E, Scardala R, Calabrese FA, Sardella G, Mancone M, Catalano C, Fedele F, Passariello R, Bogaert J and Agati L. Impact of primary coronary angioplasty delay on myocardial salvage, infarct size, and microvascular damage in patients with ST-segment elevation myocardial infarction: insight from cardiovascular magnetic resonance. *Journal of the American College of Cardiology*. 2009;54:2145-53.

18. Payne AR, Berry C, Doolin O, McEntegart M, Petrie MC, Lindsay MM, Hood S, Carrick D, Tzemos N, Weale P, McComb C, Foster J, Ford I and Oldroyd KG. Microvascular Resistance Predicts Myocardial Salvage and Infarct Characteristics in ST-Elevation Myocardial Infarction. *Journal of the American Heart Association*. 2012;1:e002246.

19. van Kranenburg M, Magro M, Thiele H, de Waha S, Eitel I, Cochet A, Cottin Y, Atar D, Buser P, Wu E, Lee D, Bodi V, Klug G, Metzler B, Delewi R, Bernhardt P, Rottbauer W, Boersma E, Zijlstra F and van Geuns RJ. Prognostic value of microvascular obstruction and infarct size, as measured by CMR in STEMI patients. *JACC Cardiovascular imaging*. 2014;7:930-9.

20. Dall'Armellina E, Karia N, Lindsay AC, Karamitsos TD, Ferreira V, Robson MD, Kellman P, Francis JM, Forfar C, Prendergast BD, Banning AP, Channon KM, Kharbanda RK, Neubauer S and Choudhury RP. Dynamic changes of edema and late gadolinium enhancement after acute myocardial infarction and their relationship to functional recovery and salvage index. *Circulation Cardiovascular imaging*. 2011;4:228-36.
